# The practice of sedation in palliative care for oncologic patients: Fantasies reported by a nursing team in a specialized hospital in Brazil: A qualitative study

**DOI:** 10.1192/j.eurpsy.2021.1977

**Published:** 2021-08-13

**Authors:** E. Turato, C. Santos, J.R. Rodrigues, A.C. Bispo, C.S. Lima

**Affiliations:** 1 Medical Psychology And Psychiatry, University of Campinas, Campinas, Brazil; 2 Lpcq - Laboratory Of Clinical-qualitative Research, University of Campinas, Campinas, Brazil

**Keywords:** nursing psychology, palliative carre, sedation in oncology, Qualitative Research

## Abstract

**Introduction:**

CONTEXTUALIZATION: Palliative sedation is a resource used to control symptoms of terminal patients in general. It is considered that it should be discussed by the professionals involved in the process, based on the competence of each one, as well as with family members and patients when possible.

**Objectives:**

AIM: To understand symbolic meanings attributed by nursing professionals who provide assistance to the terminal patient regarding to the act of the palliative sedation.

**Methods:**

Strategies: Clinical-qualitative design, semi-directed interview of open questions in depth. Nine oncologist nurses participated in the study; sample closed by the criterion of theoretical information saturation. Interviews were audio recorded, transcribed fully, categorized by qualitative content analysis. The results were discussed by colleagues of the Laboratory of Clinical Qualitative Research at the University of Campinas.

**Results:**

FINDINGS: The treatment of the data led to 6 emerging categories: (1) death maintains its ambivalent values in our culture; (2) serving the death symbolically on a tray; (3) the act of sedation and its “unfortunate coincidences”; (4) palliative sedation: agent of a pious death; (5) late sedation: cause for distress to the professional; (6) the professional’s self-comfort considering certain psychological strength from the patient and family.

**Conclusions:**

Final considerations: palliative sedation takes a general and individual meanings for the professional and even in case of experienced professionals regard to palliative sedation, the death phenomenon conduct them to expresses multiple and peculiar emotional issues, not ever perceveid.

**Disclosure:**

No significant relationships.

